# Hernia Cerebri

**Published:** 1848

**Authors:** G. W. Mears


### ARTICLE II.

*Hernia Cerebri.* Extracts from a paper read before the Indianapolis Medical Society. By G. W. Mears, M.D.

There are, as it is well known, *two* principal varieties of this disease.

The one is congenital—presenting itself in young infants before the ossification of the skull is complete.

The *other* supervenes upon the destruction of a portion of the cranium by operation with the trephine, disease, or accidental injury.

It is in relation to the last form of the second variety, or that resulting from loss of bone and dura mater by external violence, that I now propose to speak.

With regard to the pathology of the disease in question, there is a great contrariety of opinion. Some authors contend that the hernia depends upon an effusion of blood into the substance of the brain from injured vessels. Prominent among the advocates of this doctrine stands Mr. Abernethey.

Mr. Charles Bell and some others hold that the tumor is an organized fungus; and there is still another class, with Mr. Thompson at its head, who insists that the enlargement consists of a protrusion *merely* of the substance of the brain, deprived of the support of the integuments, which, in its normal state retain it "*in situ.*"

The conclusion to which a pretty thorough investigation of its nature, as well as a candid examination of the opinions held on the subject have led me is, that the tumor externally results from a determination of blood to the injured part; probably invited hither after the more violent symptoms have yielded to nature or remedial agents, in order to repair the injury. This action, if inflammatory at all, is obviously of the mildest and most passive character—perhaps more of the form of vascular turgescence or engorgement resulting, as one may imagine, rather from the vis a tergo of the heart, than from obedience to the principle "*ubi irritatio ibi fluxus*," since, in the latter case, we should look for increase of heat, pain, vascular excitement, and, perhaps, other indications of morbid excitement not here present.

Whether this hypothesis explains the phenomenon or not, it is nevertheless clear that the volume of the brain, from some cause, is increased in the vicinity of the lesion, where it finds a convenient exit; and thus we have presented to our view, some days after the removal of the bloody matter, parts of sloughed scalp, &c., &c., by the early dressings, a real hernia cerebri; a formidable looking mass of matter, corresponding in space, of course, to the opening in the cranium.

This, at one stage of its existence, is covered with a beautiful net work of healthy looking blood vessels, fixed in a very delicate membrane, which invests the protruding cerebral substance, exhibiting an instance of healthy hernia.

The treatment of this disease is properly divided into two parts. First—that which embraces the management of the wound in its earlier stages, before, and at the time sloughing takes place. I shall not detain you with this branch of the subject, as it may always be conducted on general principles, is comparatively unimportant, and in no way connected with the curative plan adopted in the second and much more important stage of the malady.

The second part of the treatment is a subject of acknowledged difficulty, and has deservedly engaged the attention of many distinguished practitioners, both in Europe and America.

Much difference of opinion must be expected to exist in regard to remedies, when views in reference to the proximate cause are so entirely discordant; and we are accordingly favored with authority for almost every kind of management we choose The mass of authority, however, seems now to be in favor of free excision of the tumor. Sir Charles Bell and Professor Gibson are among the boldest advocates of this doctrine. The latter advises large and frequent cuttings until the tumor is reduced to a level with the head.

This plan is adopted by Dr. Stewart, of Louisiana, who is the author of one of the best monographs on the subject I have met with.

Doctor Dorsey recommends *cautious* parings of the apex of the tumor; while Dr. Physic denounces excision altogether, but advises an occasional puncture with a lancet into the mass when it becomes very large, in order to reduce it by depletion. Sir Astley Cooper relies upon pressure and aqua calcis as a local application; and Baron Larry declares that the only case he ever knew cured in his life, was restored without either the use of the scalpel or local bleeding. He relies entirely upon simple, mild, local appliances.

In the case which came under my own observation, there had been considerable destruction of the substance of the brain at the time of the accident, and a small amount more came away with extravasated blood, contused and lacerated scalp, &c., with the first few dressings; the whole loss of cerebral matter up to this time amounting, perhaps, to two ounces. We employed the knife when the tumor had attained its greatest development, (say about the size of the section of a middling sized orange,) by paring from two to three lines deep, from its most prominent point. This was done as many as two or three times, removing at each operation, from one fourth to one half ounce of the substance of the enlargement, and distinguishing readily, at each cutting, the line which marked the cortical and medullary portions of the brain. The operation produced little or no inconvenience to the patient, and obviously reduced the tumor much more than the amount of substance removed by the knife—a circumstance depending, probably, upon the slight hemorrhage which attended the operation. From this period, the 27th day after the accident, the part went on to heal kindly; the brain gradually receding until it left quite a large cavity beneath the skull, thus marking the loss of substance sustained by the injury and use of the scalpel.

The little patient (about seven years old) recovered rapidly, but remained paralytic, to some extent, on the opposite side. Now, whether this is the effect of the original injury entirely, or results in part from the practice in the case, is an interesting inquiry. Certain is is, that a large portion of the brain is lost by the application of the scalpel, and I am not prepared to say whether such results will not uniformly follow its free use. Doct. Stewart removed, at a single cutting, nearly half the tumor in the case he reports, but he was greatly alarmed at first by the hemorrhage, and then by a train of the most distressing nervous symptoms, which required a free use of stimulants to overcome. Still he cured his case, and now ranks among the warm advocates of the scalpel.

But, notwithstanding all these favorable results, we have, on the other side of the question, the successful management of the disease without the operation; and when we hear such names as Physic, Cooper, and Larrey, with the potent argument of the revolting nature of the operation—and the additional and weighty fact that we are cutting away a part of a vital organ which may result in future injury to our patient, I think it becomes us to hesitate, at least, and examine well all chances for recovery by other means before we resort to excision.

My impression is, that when the tumor presents an elastic, healthy appearance and is covered by a vascular net work, which I presume, is generally the case in healthy subjects, it will be perfectly safe to trust the case to the light, cool, and unirritating appliances of Larrey, with appropriate general treatment. By this mild management, (which may scarcely be dignified with the name of remedy, since it is a mere quiet waiting for nature to do its work)—many cases, as we are assured on the best authority, are cured. The tumor, after acquiring a certain size, recedes and takes part in the common offices of the brain. Now, if the point is established that cases are cured without the aid of the knife; or, in other words, that the tumor retracts after a certain period of its existence, taking its former place within the skull, and then again performing its part in the function of the brain, it is very obvious to me that the position I have taken in regard to its pathology is, to say the least, tenable—It goes certainly to prove that in healthy subjects the presence of the hernia is itself an evidence that nature has commenced its curative process, and if left undisturbed by officious surgery will, in most cases, soon effect a perfect restoration, of the diseased part.

We are here presented with one of those beautiful and ever recurring instances of the recuperative energies of nature, in which she so frequently displays the riches of her resources, and often causes human skill to blush on account of the superior evidence of her councils.

